# Lung function trajectories in children with post-prematurity respiratory disease: identifying risk factors for abnormal growth

**DOI:** 10.1186/s12931-021-01720-0

**Published:** 2021-05-10

**Authors:** Jonathan C. Levin, Catherine A. Sheils, Jonathan M. Gaffin, Craig P. Hersh, Lawrence M. Rhein, Lystra P. Hayden

**Affiliations:** 1 Division of Newborn Medicine, Boston Children’s Hospital, 300 Longwood Ave Hunnewell 4, Boston, MA 02115 USA; 2 Division of Pulmonary Medicine, Boston Children’s Hospital, Boston, MA USA; 3 Channing Division of Network Medicine, Brigham and Women’s Hospital, Boston, MA USA; 4 Department of Pediatrics, University of Massachusetts, Worcester, MA USA

**Keywords:** Pulmonary function tests, Bronchopulmonary dysplasia, Chronic lung disease of prematurity

## Abstract

**Background:**

Survivors of prematurity are at risk for abnormal childhood lung function. Few studies have addressed trajectories of lung function and risk factors for abnormal growth in childhood. This study aims to describe changes in lung function in a contemporary cohort of children born preterm followed longitudinally in pulmonary clinic for post-prematurity respiratory disease and to assess maternal and neonatal risk factors associated with decreased lung function trajectories.

**Methods:**

Observational cohort of 164 children born preterm ≤ 32 weeks gestation followed in pulmonary clinic at Boston Children’s Hospital with pulmonary function testing. We collected demographics and neonatal history. We used multivariable linear regression to identify the impact of neonatal and maternal risk factors on lung function trajectories in childhood.

**Results:**

We identified 264 studies from 82 subjects with acceptable longitudinal FEV_1_ data and 138 studies from 47 subjects with acceptable longitudinal FVC and FEV_1_/FVC data. FEV_1_% predicted and FEV_1_/FVC were reduced compared to childhood norms. Growth in FVC outpaced FEV_1_, resulting in an FEV_1_/FVC that declined over time. In multivariable analyses, longer duration of mechanical ventilation was associated with a lower rate of rise in FEV_1_% predicted and greater decline in FEV_1_/FVC, and postnatal steroid exposure in the NICU was associated with a lower rate of rise in FEV_1_ and FVC % predicted. Maternal atopy and asthma were associated with a lower rate of rise in FEV_1_% predicted.

**Conclusions:**

Children with post-prematurity respiratory disease demonstrate worsening obstruction in lung function throughout childhood. Neonatal risk factors including exposure to mechanical ventilation and postnatal steroids, as well as maternal atopy and asthma, were associated with diminished rate of rise in lung function. These results may have implications for lung function trajectories into adulthood.

**Supplementary Information:**

The online version contains supplementary material available at 10.1186/s12931-021-01720-0.

## Introduction

Survivors of prematurity are at risk for respiratory morbidity and abnormal lung function throughout life [1]. Post-prematurity respiratory disease, including bronchopulmonary dysplasia (BPD), may be a progenitor to adult chronic obstructive pulmonary disease [2–5]. BPD affects up to 40% of former preterm infants born at < 28 weeks gestation, with about 10,000 new cases of BPD in the United States every year [6, 7]. Of survivors with BPD, more than 50% may have abnormal lung function when they are of school age [8]. Pulmonary function in survivors with BPD often demonstrates an obstructive pattern, specifically with regard to decreased forced expiratory volumes in one second (FEV_1_) and decreased ratio of FEV_1_ to Forced Vital Capacity (FVC) [8–10]. Adulthood survivors of prematurity demonstrate ongoing obstructive abnormalities that persist beyond childhood [11–13].

Infants born preterm since the early 1990s, when surfactant, antenatal steroids, and lung-protective ventilation became standards of care, are just reaching young adulthood [14]. Longitudinal follow-up among survivors of prematurity with ongoing lung disease is becoming increasingly important to understand the long-term outcomes and healthcare needs in this population [15, 16]. Classically, recovery of lung function from prematurity is believed to be possible with ongoing alveolar growth through childhood, in the absence of ongoing pulmonary insults [17]. However, few studies have addressed trajectories of lung function and risk factors for abnormal growth.

Survivors of prematurity may have an abnormal growth in pulmonary function as evidence by diminished rate of rise during childhood and adolescence, abnormal peak lifetime function, and a more rapid decline in lung function in adulthood [18]. Available data of serial measurements of pulmonary function in this population suggest worsening airway obstruction over time in children with a diagnosis of BPD [19–24]. However, to our knowledge pulmonary function trajectories have not been reported in a United States based cohort. Furthermore, few contemporary studies focus on identifying risk factors from the neonatal and early childhood course associated with abnormal pulmonary function trajectories in childhood.

We aimed to characterize lung function trajectories in a contemporary observational cohort of children with post-prematurity respiratory disease followed in pulmonary clinic at Boston Children’s Hospital. Our objectives were to (1) describe childhood spirometry in a cohort of children born preterm with chronic respiratory disease, (2) measure changes in spirometry values over time in children with serial lung function measurements, and (3) identify risk factors from the neonatal course associated with decreased lung function trajectories. We hypothesized that survivors of prematurity with respiratory disease would demonstrate an obstructive pattern of pulmonary function, and that those with more severe disease in the neonatal period would demonstrate less potential for improvement in lung function during childhood.

## Methods

### Subjects

We selected subjects who performed spirometry from a patient registry of former preterm infants born ≤ 32 weeks gestational age (GA) followed at the Boston Children’s Hospital Preterm Lung Patient Registry between 2008 and 2019 (March 29, 2019 lung function dataset). The patient registry includes former preterm infants followed by pulmonology for post-prematurity respiratory disease and is approved by the institutional review board at Boston Children’s Hospital. Informed consent was obtained for each participant enrolled after 2008; an additional 38 subjects were enrolled under a waiver of consent for retrospective review. Excluded are those with known cyanotic cardiac lesions, genetic syndromes, or congenital malformations that contribute to their respiratory disease. Subjects were seen as clinically indicated, typically at least every 3 months in the first year of life, every 6 months in the second year, and annually thereafter; more frequent visits are as indicated based on symptoms and clinical course.

### Measurements

Demographics, neonatal intensive care unit (NICU) history, and maternal information were collected after enrollment including questionnaire data collected at clinic visits. Details of the NICU course were obtained from NICU discharge summaries. BPD was defined based upon modified National Heart Lung and Blood Institute (NHLBI) criteria as having used at least 28 days of oxygen after birth with severity assessed based on respiratory support at 36 weeks post-menstrual age [25]. Respiratory support at 36 weeks and at discharge was defined as nasal cannula oxygen, positive pressure, or mechanical ventilation. Subjects without an available BPD severity were included in the overall lung function analyses but removed from analyses subset on BPD severity. Tobacco exposure, both during pregnancy and secondhand exposure in the home, was based on questionnaires collected as part of the patient registry during clinic visits. Maternal asthma, hay fever, and eczema were self-reported; atopy was defined as having at least one of these diagnoses. When available, follow-up questionnaires completed in the first three years of life were used, which included data on respiratory illnesses (defined as bronchiolitis, reactive airway disease, asthma flare, BPD flare, or pneumonia), medication use, and palivizumab and influenza vaccination. These questionnaires were administered up to every 6 months in frequency, and responses over the first three years were aggregated such that as ‘yes’ response on any individual questionnaire was recorded as a ‘yes’ overall.

Subjects performed pre-bronchodilator spirometry as clinically indicated according to American Thoracic Society Guidelines on a Medisoft SpiroAir® machine (Sorinnes, Belgium); data were recorded in and extracted from Morgan Scientific ComPAS software (Haverhill, MA, USA) [26]. Spirometry was normalized using Global Lung Function Initiative (GLI) equations to obtain percent predicted values [27]. Each test was visually assessed using modified pediatric American Thoracic Society criteria, including acceptability of FEV_1_ and FVC (see Additional file 1). Studies were excluded completely if they did not meet criteria for FEV_1_ on at least one effort; FVC data was only included from studies that had acceptable end-effort criteria. We selected year’s best pre-bronchodilator spirometry efforts for FEV_1_% predicted, FVC % predicted, and FEV_1_/FVC ratio for each year the subject completed spirometry to avoid bias of testing obtained during time of illness. Post bronchodilator data were obtained based on clinical indication, and a greater than 10% change was considered a positive response. Pulmonary function trajectories were assessed in subjects with at least two pre-bronchodilator spirometry efforts at different ages.

### Statistical analysis

Primary analysis was performed using a two staged model for lung function trajectories [28]. This two-staged model was preferred over a linear mixed effects model as to give each subject equal weighting; subjects with more measurements could otherwise bias the model towards those with more severe disease, who may have had more frequent  lung function measurements. In the first stage, linear models were used to calculate the change for each subject in FEV_1_% predicted / year, FVC % predicted / year, and FEV_1_/FVC (absolute) / year. In the second stage, the coefficient from each subjects’ individual model was used as the outcome variable in a multivariable linear regression to examine effects of neonatal exposures and change in pulmonary function over time. This regression was controlled for GA, birthweight (BW), and first spirometry value. As height, age, race and gender are incorporated in percent predicted values, we did not adjust for these characteristics separately in multivariable analyses. We used a significance level of p < 0.05. Results were reported as absolute differences (β) with 95% confidence intervals. Subjects with missing data were removed from specific analyses. Analysis was performed using R v4.0.2 (2020-06-22) with packages tidyverse v1.3, lme4 v1.1.23, ggplot2 v3.3.2, and rspiro v0.2.

### Sensitivity analyses

A sensitivity analysis was performed using linear mixed effect models with pulmonary function as the outcome, using subject and age as random effects, and using neonatal exposures, GA, BW, age, and the interaction term of (neonatal exposure * age) as fixed effects [24].

Given that most available spirometry in this cohort was performed at ≤ 12 years of age, a separate sensitivity analysis only using these data was performed, removing those studies performed after this point from analysis.

## Results

**Fig. 1:**
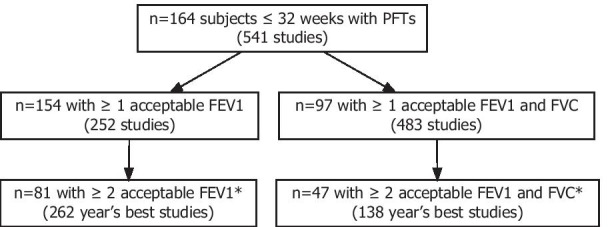
Study flow diagram. *Studies performed at different ages

We identified 164 former preterm infants ≤ 32 weeks GA with pulmonary function testing (Fig. 1). These subjects were predominantly Caucasian, with a slight male predominance (54%). Median GA was 26 1/7 weeks with median BW 877 g. The plurality of subjects had moderate to severe BPD, with a high degree of respiratory and other neonatal morbidities, including need for surfactant, postnatal steroid exposure in the NICU, patent ductus arteriosus (PDA) ligation, gastrostomy tube placement, and respiratory support at 36 weeks post-menstrual age (Table 1). Self-reported tobacco exposure was low. There were high rates of self-reported maternal atopy. Follow-up data over the first three years of age were available for 42 infants; nearly half required an emergency department visit or hospitalization for respiratory illness, and one-third developed a lower respiratory tract infection. Though subjects were born over a wide timespan (1999–2014), using linear or logistical regression with birth year as a covariate there was no evidence for change over time for GA, BW, surfactant, antenatal steroids, ventilated or CPAP days, PDA diagnosis, PDA ligation, necrotizing enterocolitis, gastrostomy, and moderate/severe BPD. Over time, tracheostomy placement decreased (p = 0.03) as did postnatal steroid use (p = 0.004).

**Table 1:** Subject characteristics

	N = 164
*Demographics*	
Median birth year, (range)	2008 (1999–2014)
Males, no. (%)	88 (54%)
Race, no. (%)	
White	129 (79%)
Black	14 (9%)
Asian	2 (1%)
Other	19 (11%)
Hispanic, no./total (%)	19/151 (13%)
*Maternal history*	
Median maternal age (IQR)	33 (29.25—37)
Maternal asthma, no./total (%)	38/105 (36%)
Maternal hay fever, no./total (%)	31/105 (30%)
Maternal eczema, no./total (%)	26/106 (25%)
Maternal atopy, no./total (%)	60/106 (57%)
*Neonatal history*	
Median gestational age, weeks (range)	26 1/7 (23 3/7—32)
Median birth weight, grams (range)	813 (455–1927)
Small for gestational age, no. (%)	13 (8%)
Multiple gestation, no. (%)	67/163 (41%)
Bronchopulmonary dysplasia, no. (%)	
None	16 (9%)
Mild	15 (10%)
Moderate	49 (30%)
Severe	68 (41%)
Unclassified	16 (10%)
Antenatal corticosteroid (any), no. (%)	114/136 (84%)
Antenatal steroid (complete), no. (%)	83/105 (78%)
Median ventilated days (IQR)	31 (8–49)
Median CPAP days (IQR)	25 (10–40)
Surfactant, no./total (%)	138/142 (97%)
PDA, no./total (%)	117/150 (78%)
PDA ligation, no./total (%)	50/148 (34%)
NEC, no./total (%)	25/148 (17%)
Intraventricular hemorrhage, no./total (%)	
Grade I/II	25/97 (27%)
Grade III/IV/periventricular leukomalacia	7/97 (7%)
Gastrostomy tube, no./total (%)	42/142 (30%)
Tracheostomy, no./total (%)	14/145 (10%)
Postnatal steroids (NICU), no./total (%)	41/106 (33%)
Breastmilk nutrition at discharge no./total (%)	
Exclusive breastmilk	39/150 (26%)
Mixed	54/150 (36%)
Formula	57/150 (38%)
Resp support or O_2_ at 36 wk, no./total (%)	117/149 (79%)
Resp support or O_2_ at discharge, no./total (%)	80/160 (50%)
*Tobacco exposure*	
Maternal tobacco use, no./total (%)	3/107 (3%)
Secondhand smoke exposure in home, no./total (%)	11/164 (7%)
*Follow-up 0–3 years (n = 42 with data)*	
Received palivizumab	25/32 (60%)
Received flu shot	36/42 (86%)
ER or hospitalization for respiratory cause	19 (45%)
Hospitalization for respiratory cause	7 (17%)
Lower respiratory tract infection	14 (33%)
*Spirometry*	
Mean age at spirometry, years (range)	6.7 (3–20)
Mean number of studies per subject, (range)	3.3 (1–18)

*IQR* Interquartile range, *BW*  birthweight, *GA*   gestational age, *PDA*  patent ductus arteriosus, *NEC*  necrotizing enterocolitis. When a distinct total is listed, there was missing data not included in analysis. Lower respiratory tract infection defined as bronchiolitis, asthma flare, reactive airway disease, BPD flare, or pneumonia.

### Pulmonary function studies

**Fig. 2:**
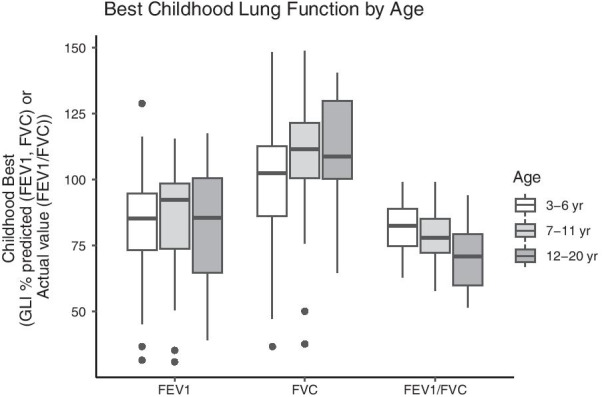
Best pulmonary function over childhood and adolescence. Error bars represent median, 25th and 75th percentile. There were no significant differences between groups

Subjects performed 541 distinct spirometry sessions, with an average age of six years at testing and an average of three sessions per subject. There were 483 tests (89%) from 155 subjects (94%) with acceptable FEV_1_ data and 252 tests (47%) from 98 subjects (59%) with both acceptable FEV_1_ and FVC data (Fig. 1). Best FEV_1_, FVC, and FEV_1_/FVC were reduced at ages 3–6 years, 7–11 years, and 12 + years compared to normative values (Table 2, Fig. 2).

**Table 2:** Best pulmonary function over childhood and adolescence

	3–6 years	7–11 years	12–20 years
*Best FEV1 (% predicted)*			
n	128	71	14
mean (s.d.)	83.6 (17)	87.1 (18)	82.6 (25)
*Best FVC (% predicted)*			
n	60	55	12
mean (s.d.)	100 (21)	110 (20)	111 (21)
*Best FEV1/FVC*			
n	60	55	12
mean (s.d.)	82 (9.1)	79 (8.9)	70.6 (14)

Post-bronchodilator testing was performed in 94 tests from 57 subjects in this sample; mean percent change in FEV_1_ was 12.9% (s.d. 14.3). There were 50 tests (53%) that demonstrated a positive response to bronchodilator (as defined by change in FEV_1_ ≥ 10%); 38 subjects (67%) demonstrated a positive response to bronchodilator in at least one test.

### Pulmonary function trajectories

Longitudinal FEV_1_ data were available for 81 subjects, totaling 262 year’s best studies performed between 3 and 20 years of age (median 6.0 years). Serial FVC and FEV_1_/FVC data were available for 47 subjects, totaling 138 studies performed between 4 and 20 years of age (median 7.0 years).

**Fig. 3:**
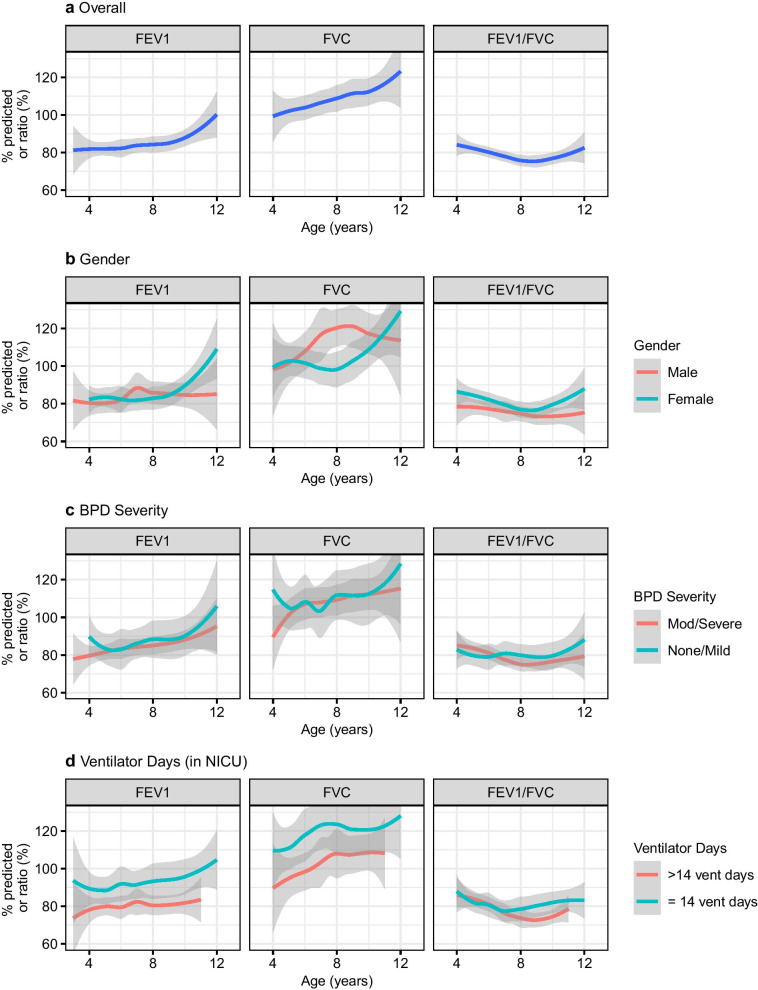
Spirometry trends for study population through age 12. Lines represent best fit trend over time by locally estimated scatterplot smoothing; grey area is 95% CI. BPD Severity by NHLBI Workshop Criteria (O2 for 28 days + respiratory support at 36 weeks)

Overall, FEV_1_% predicted and FVC % predicted increased over time, suggesting some degree of ‘catch-up’ growth. However, growth in FVC % predicted (mean 2.7%, s.d. 6.1% predicted per year) outpaced growth in FEV_1_% predicted (mean 1.8%, s.d. 5.8% predicted per year), resulting in an FEV_1_/FVC that declined over time (mean − 1.1%, s.d. 3.7% predicted per year), suggesting a more obstructive pattern. Rate of rise of FEV_1_% predicted was lower for Black (p = 0.03) and Northeast Asian (p = 0.002) subjects, and rate of rise of FEV_1_/FVC was lower for Northeast Asian subjects (p = 0.001); otherwise rate of change in pulmonary function was not different based on gender or BPD severity by univariate linear regression (Table 3). Data of children ≤ 12 years of age at the time of testing suggest a nadir of FEV_1_/FVC at 8–9 years (Fig. 3a).

**Table 3:** Lung function trajectories

	FEV1% predicted (%/year)	FVC % predicted (%/year)	FEV1/FVC (/year)
Overall	1.8 ± 5.8	2.7 ± 6.1	− 1.1 ± 3.7
Gender			
Male (ref)	1.8 ± 5.9	3.3 ± 5.9	− 0.5 ± 4.3
Female	1.9 ± 5.8	2.2 ± 6.3	− 1.5 ± 3.3
Race			
Caucasian (ref)	2.7 ± 5.7	2.5 ± 6.4	− 0.46 ± 3.3
Black	− 1.7 ± 5.9*	5 ± 6.4	− 2.9 ± 5.2
Northeast Asian	− 6.9 ± 5.3*	3.3 ± NA	− 12 ± NA*
Other	1.3 ± 3.6	1.8 ± 4.4	− 1.2 ± 1.4
BPD severity			
None/mild (ref)	2.9 ± 4.9	3.6 ± 6.8	0.11 ± 3.8
Moderate/severe	1.3 ± 6.2	2.5 ± 6.1	− 1.8 ± 3.7

Values represent mean increase ± s.d. *p < 0.05. Significance determined by univariate linear regression. *BPD* Bronchopulmonary Dysplasia, severity defined by modified 2001 NHLBI consensus definition. NA listed for s.d. when only single result available

There was no change in pulmonary function trajectories over time by birth year (FEV1% predicted p = 0.8, FVC % predicted p = 0.5, FEV1/FVC p = 0.9 by linear regression).

### Neonatal risk factors associated with abnormal lung function trajectories

In multivariable analyses using linear regression, controlling for GA, BW, and first spirometry value, longer duration of mechanical ventilation was associated with a lower rate of rise in FEV_1_% predicted and greater decline in FEV_1_/FVC. Postnatal steroid exposure in the NICU was associated with lower rate of rise in FVC % predicted. Tracheostomy was associated with a greater decline in FEV_1_/FVC; gastrostomy was associated with lower rate of rise in FEV_1_% predicted. Surgical ligation of a PDA was associated with a greater improvement in FEV_1_/FVC over time. Maternal asthma and atopy were associated with lower rate of rise in FEV1% predicted; and maternal asthma was associated with a greater decline in FEV_1_/FVC (Table 4).

**Table 4:** Predictors of pulmonary function growth

	FEV1% predicted Δ / year	FVC % predicted Δ / year	FEV1/FVC Δ / year
*Neonatal*			
GA (1 week)	− 0.0038 (− 0.95, 0.95)	1 (− 0.28, 2.4)	− 0.14 (− 0.97, 0.69)
BW (100 g)	0.22 (− 0.43, 0.87)	− 0.071 (− 0.94, 0.8)	0.33 (− 0.19, 0.85)
SGA	− 2.4 (− 7.6, 2.9)	− 1.1 (− 7.5, 5.3)	− 1.2 (− 5.1, 2.7)
Multiple gestation	− 0.00061 (− 2.7, 2.7)	− 2.5 (− 6.3, 1.3)	1 (− 1.4, 3.5)
Antenatal steroids (any)	− 1.6 (− 6, 2.8)	1.3 (− 8.3, 11)	− 3.2 (− 8.6, 2.2)
Antenatal steroids (complete)	1.7 (− 2.6, 5.9)	1 (− 5.4, 7.4)	0.45 (− 3, 3.9)
Surfactant	− 0.77 (− 9.5, 7.9)	4.8 (− 8.2, 18)	− 5.2 (− 13, 2.5)
Ventilated days (1 day)	− 0.026 (− 0.045, − 0.0074)*	− 0.011 (− 0.037, 0.014)	− 0.017 (− 0.03, − 0.0047)*
CPAP days (1 day)	0.011 (− 0.068, 0.09)	0.053 (− 0.05, 0.16)	− 0.026 (− 0.09, 0.038)
PDA	0.29 (− 3.3, 3.9)	− 3.6 (− 8.8, 1.7)	0.6 (− 2.5, 3.7)
PDA ligation	− 0.17 (− 3.1, 2.8)	− 2.1 (− 6.2, 2.1)	3.2 (1.1, 5.3)*
NEC	2.8 (− 0.98, 6.6)	3.3 (− 1.5, 8.1)	1.2 (− 1.7, 4.2)
Severe IVH	− 2.3 (− 9.1, 4.4)	− 2 (− 8.5, 4.5)	− 0.66 (− 6.1, 4.8)
Postnatal steroids (in NICU)	− 2.8 (− 6, 0.44)	− 3.9 (− 7.3, − 0.38)*	− 1.8 (− 5.2, 1.6)
Gastrostomy tube	− 3.4 (− 6.5, − 0.3)*	− 1.2 (− 4.9, 2.4)	0.26 (− 2.4, 2.9)
Tracheostomy	− 4.5 (− 9.3, 0.36)	− 4.7 (− 11, 1.6)	− 4.8 (− 8.3, − 1.3)*
Discharge off breast milk	− 0.55 (− 2.3, 1.2)	1.2 (− 1.3, 3.7)	− 0.19 (− 1.8, 1.4)
Any resp support at 36 weeks	− 2.1 (− 5.8, 1.5)	0.59 (− 4.1, 5.2)	− 1.4 (− 4.4, 1.5)
Any resp support at discharge	− 0.028 (− 2.9, 2.9)	1.3 (− 2.8, 5.3)	− 2.5 (− 4.9, 0.021)
*Maternal history*			
Age	0.16 (− 0.13, 0.45)	0.017 (− 0.38, 0.41)	− 0.008 (− 0.23, 0.21)
Asthma	− 3.2 (− 6, − 0.37)*	− 0.75 (− 5, 3.5)	− 2.1 (− 4.6, 0.36)
Eczema	− 0.13 (− 4.1, 3.8)	0.49 (− 4.5, 5.5)	1.9 (− 1.2, 4.9)
Hay fever	− 0.46 (− 3.9, 3)	− 2.6 (− 7.3, 2)	− 1.4 (− 4.2, 1.3)
Atopy	− 2.9 (− 5.7, − 0.13)*	− 2.1 (− 6.2, 2.1)	− 0.33 (− 2.8, 2.2)
Secondhand smoke exposure at home	1.3 (− 3.7, 6.2)	0.89 (− 5, 6.8)	0.57 (− 3, 4.2)
*Follow-up (0–3 year) history (n = 42)*			
Received palivizumab	− 1.7 (− 13, 9.3)	− 1.3 (− 20, 17)	− 4 (− 15, 6.5)
Received flu vaccination	− 2.8 (− 12, 6.9)	− 0.74 (− 15, 13)	− 2.8 (− 13, 7)
Any ED Visit/hospitalization	2.1 (− 6.4, 11)	− 1.5 (− 16, 13)	0.76 (− 8.5, 10)
Any hospitalization	− 11 (− 23, 1.7)	− 5.3 (− 41, 30)	0.89 (− 12, 14)
Any lower respiratory tract infection	5.2 (− 3.5, 14)	− 10 (− 25, 3.8)	0.74 (− 14, 16)

Multivariable analysis using two staged linear model, controlling for birthweight (*BW*), gestational age (*GA*), and first measured spirometry value. Effect β represents, controlling for above, change in lung function outcome per unit change in risk factor. *p < 0.05. *SGA* Small for gestational age; *PDA* patent ductus arteriosus; *NEC* necrotizing enterocolitis. When unit is listed next to risk factor, it indicates unit change for corresponding effect size on spirometry result

### Sensitivity analyses

Sensitivity analysis using a linear mixed effects model as opposed to the two staged linear regression revealed similar results in the effect of longer duration of mechanical ventilation associated with a lower rate of rise in FEV_1_% predicted and greater decline in FEV_1_/FVC. The linear mixed model differed from the primary two staged model in that antenatal steroids were associated with a lower rate of rise in FEV_1_% predicted, maternal asthma was associated with a lower rate of rise of FEV_1_/FVC, and multiple post-hospital exposures were associated with lower pulmonary function trajectories (see Additional file 2).

A separate sensitivity analysis included only spirometry from age at testing ≤ 12 years. We found similar rate of rise in FEV_1_ and FVC % predicted (2.1%/year and 2.9%/year, respectively) and similar decline over time in FEV_1_/FVC (-1.0%/year). In this subset of pulmonary function studies, we found similar neonatal and maternal predictors of pulmonary function trajectories as the main analysis (see Additional file 3).

## Discussion

We have demonstrated that in a cohort of U.S. born children followed longitudinally in pulmonary clinic for post-prematurity respiratory disease, average lung function throughout childhood was reduced, and lung function trajectories were abnormal with patterns becoming more obstructive over time. FEV_1_ and FEV_1_/FVC throughout childhood and adolescence were lower compared to GLI reference standards in healthy children [27]. Just over half of efforts with post-bronchodilator data showed significant improvement after bronchodilator administration; two-thirds of subjects with at least one post-bronchodilator effort demonstrated improvement an at least one effort, consistent with prior cohorts [9]. Number of ventilated days, gastrostomy tube placement, maternal atopy, and maternal asthma were associated with diminished rise in FEV_1_% predicted over time; postnatal steroid exposure was associated with diminished rise in FVC % predicted over time; and number of ventilated days and tracheostomy were associated with a greater decrease in FVC % predicted.

Post-neonatal exposures and events in the first years of life, including lower respiratory tract infections, rehospitalization, and RSV prophylaxis were not associated with pulmonary function trajectories later in childhood, though number of subjects with data available was smaller. Nonetheless, for RSV prophylaxis this is consistent with previous studies [29, 30].

Among subjects with serial spirometry, pulmonary function trajectories became more obstructive over time. While there seemed to be ‘catch-up’ growth in FEV_1_% predicted and FVC % predicted in this cohort, FVC growth outpaced FEV_1_, resulting in a declining FEV_1_/FVC over time. This pattern may suggest dysanaptic growth, or differential growth of the airways and lung parenchyma [31]. Both worsening obstruction and dysnapatic growth have been suggested in previous longitudinal cohorts of former preterm infants [19–24, 32–34]. Studies from the GLI have suggested that in healthy children there is a slight decrease in FEV_1_/FVC between ages 6 and 11 years, of about − 1.5 per year overall, and less so in Caucasian children (− 0.67 per year) and in females (− 1.2 per year) [35]. In this study, change per year in FEV_1_/FVC was similar (− 1.1 per year); we also found a trend toward greater decreases in African Americans (− 2.9 per year versus − 0.5 per year in Caucasians) like previous reports, but also a trend toward greater decreases in females (− 1.5 per year versus − 0.5 per year in males), which for gender is the opposite of what was shown by GLI in healthy children. Even with similar rates of change, it is notable that in this study children started with lower and more obstructed lung function, such that the dysanaptic growth that occurred resulted in worsening obstruction over time. Additionally, the data suggest an earlier nadir of 8–9 years than has been reported in healthy children (10–11 years).

To our knowledge, this is the first report of abnormal pulmonary function trajectories in a U.S. born cohort of children with post-prematurity respiratory disease in the post-surfactant era. Here we demonstrate similar findings of worsening obstruction over time. Of note, this cohort, which includes children followed in pulmonary clinic for respiratory disease related to prematurity, had higher rates of BPD, higher durations of mechanical ventilation, and greater postnatal corticosteroid use than other previously described post-surfactant era cohorts. Thus, this is more representative of a population with significant respiratory morbidity than prematurity, rather than a cohort of children born preterm overall. In the described cohort here, we demonstrate worsening airway obstruction among a population of former preterm infants with respiratory disease seeking medical follow-up who have the greatest need for catch up growth.

Our study also identifies risk factors in the neonatal period associated with more abnormal lung function trajectories. A recent study on an earlier cohort (1997 to 2003) in Australia also reported abnormal pulmonary function trajectories through childhood in former preterm infants, and identified earlier gestation, exposure to tobacco smoke, and greater duration of neonatal oxygen supplementation as risks for a more rapid rate of decline [24]. In our cohort, few mothers self-reported tobacco use or household exposure, suggesting underreporting. Results from these retrospective cohorts, however, do not resolve whether time on oxygen or mechanical ventilation during the neonatal period is simply an early marker of more severe pulmonary disease or is causative, through mechanisms of oxygen free radical toxicity, barotrauma, and volutrauma [36].

Our findings have implications for the trajectory of lung function in this population into adulthood. Given that children born preterm likely continue to have abnormal pulmonary growth throughout childhood raises concern that even though alveolar growth continues past the newborn period into childhood and even adolescence, these patients may not “catch up” with healthy controls with regard to pulmonary function and respiratory capacity. Due to ongoing abnormal lung growth, former preterm infants may be unable to achieve maximum normal lifetime lung function in early adulthood. Former preterm infants may also be susceptible to an earlier decline in lung function during adulthood, leading to patients with lung function that meets diagnostic criteria for Chronic Obstructive Pulmonary Disease (COPD) in early adulthood despite avoiding of toxic exposures such as cigarette smoking [24].

This is a prospective observational study that only captures subjects followed in pulmonary clinic and therefore is most applicable to the clinical population at pediatric tertiary care centers where patients with post-prematurity respiratory disease are followed and may not be generalizable to all children born preterm. Study strengths include the cohort size, the well-phenotyped population combining neonatal course details with serial spirometry data, and the recency of the cohort, enhancing external validity. Childhood environmental and infectious exposures may further affect lung function growth in this population, as has been shown in longitudinal population cohorts [37]. More extensive tracking post-neonatal symptoms exposures in early childhood will be included in future studies to assess how such exposures modify pulmonary function trajectories.

Our study population at baseline had a high degree of neonatal respiratory and non-respiratory morbidity [38]. This may have led to a selection bias, particularly among individuals with no BPD or mild BPD, to represent a population with lower pulmonary function at baseline. Notably, children unable to perform pulmonary function tests (PFTs) due to severe cognitive or neurological impairment are excluded. In addition, BPD severity was not available for 10% of subjects, based on limitations in documentation of oxygen use at 36 weeks and a physiologic test of respiratory support needs at 36 weeks was not routinely documented in NICU discharge summaries [39]. Given this, differences (or lack thereof) between different levels of BPD severity should be interpreted with caution. We also did not have a control population to serve as a reference standard with PFTs, and instead used internationally acceptable GLI scores to compare our cohort to a standard reference population of children. As this cohort is captured only during clinically indicated visits it is lacking ideal standardized follow-up schedule for pulmonary function. To account for this, we created linear models based on serial PFT data points to extrapolate trajectory through childhood. Although this approach has limitations, the ideal growth of pulmonary function is relatively steady during early childhood [24]. The relatively few subjects in the cohort with multiple PFTs could lead to bias toward children and those with greater illness severity. We attempted to account for those children who required multiple testing in one year by only using the year’s best lung function in analyses, thus minimizing the chance for confounding the results from including sick visit PFTs. However, it is possible that the only spirometry values for a given year for an individual subject may have been in the context of a sick visit. We had limited data on maternal educational and economic status which are known to associate with pulmonary outcomes of prematurity, though may not contribute as much as maternal race, which was included [40]. We also had relatively few subjects with testing above 12 years of age, however in a sensitivity analysis only including results up until 12 years, the main outcomes did not change. Ideally, we would have had pre- and post-bronchodilator testing on all available subjects, but given the fact that is a clinical cohort we only have them available in subjects as clinically indicated, which limits their interpretation and likely biases the findings towards the positive association with bronchodilator response.

## Conclusions

We found that former preterm infants with chronic lung disease of prematurity are at risk for reduced lung function as well as decreased trajectories of lung function growth. Neonatal risk factors including exposure to mechanical ventilation and postnatal steroids, as well as maternal atopy and asthma, were associated with ongoing poor growth in lung function throughout childhood. There is a need to better understand the lifetime trajectory of pulmonary function in former preterm infants as well as the factors associated with potential to achieve catch-up growth and to ultimately reach a normal expected maximal lung function in early adulthood. Our study suggests that despite ongoing alveolar growth past the neonatal period, lung function growth remains diminished in childhood in this cohort of former preterm infants with chronic lung disease, which may have implications for the trajectory of their lung function into adulthood putting them at risk for developing COPD.

## Supplementary Information

**Additional file 1: **Lung function scoring.

**Additional file 2: **Predictors of pulmonary function trajectories using alternative linear mixed effects model.

**Additional file 3: **Pulmonary function trajectories among studies ≤ 12 years age.

## Data Availability

The datasets analyzed during the current study are not publicly available due to individual privacy considerations, but are available from the corresponding author on reasonable request.

## Abbreviations

BPD: Bronchopulmonary dysplasia
BW: Birthweight
COPD: Chronic obstructive pulmonary disease
FEV_1_: Forced expiratory volume in one second
FVC: Forced vital capacity
GA: Gestational age
GLI: Global lung function initiative
NICU: Neonatal intensive care unit
PFTs: Pulmonary function tests
