# Supplementary material for: Lung function trajectories in children with post-prematurity respiratory disease: identifying risk factors for abnormal growth
Source: Respir Res. 2021 May 10;22:143. doi: 10.1186/s12931-021-01720-0 (PMC8112031; doi:10.1186/s12931-021-01720-0)
Supplement: Supplementary file 1 — Additional file 1: Lung function scoring. [file 12931_2021_1720_MOESM1_ESM.doc]

**TITLE:** Lung Function Trajectories in Children with Post-Prematurity Respiratory Disease: identifying risk factors for abnormal growth

**AUTHORS:**Levin, Jonathan C. MD ^1,2^; Sheils, Catherine A. MD ^2^; Gaffin, Jonathan M. MD MMSc^2^; Hersh, Craig P. MD MPH^3^; Rhein, Lawrence M. MD MPH^4^; Hayden, Lystra P. MD MMSc^2,3^

*Appendix 1. Lung Function* Scoring

PFTs were scored based on ATS guidelines. Maximum 6 points were given for effort: (1) rapid rise of flow-volume loop, (2) discernable peak of flow-volume loop, (3) smooth descending limb of flow-volume loop, (4) smooth termination of flow-volume loop, (5) plateau of volume time curve, and (6) minimum 3 second effort on volume time curve. PFTs were also scored for reproducibility, with a maximum 2 points: (1) 3 acceptable efforts, and (2) FEV_1_ within 10% or 0.1-0.15 L of each other. Tests were scored by at authors (J.L., J.G., L.H) and if there was a questionable score, a second scorer reviewed the study. We included a spirometry effort for analysis by the following criteria: for forced expiratory volume in one second (FEV_1_), a score >2 for the initial forced expiratory effort (rapid rise, peak, and smooth descending limb of flow-volume loop); for forced vital capacity (FVC), a score of > 5 for effort throughout forced expiration (rapid rise, peak, smooth descending limb, and smooth termination of flow-volume loop; and plateau and minimum 3 second effort on volume time curve). We did not use reproducibility criteria for inclusion in this study given that most studies were formed by young children and we were only using the single best effort for analysis.
