# Supplementary material for: Lung function trajectories in children with post-prematurity respiratory disease: identifying risk factors for abnormal growth
Source: Respir Res. 2021 May 10;22:143. doi: 10.1186/s12931-021-01720-0 (PMC8112031; doi:10.1186/s12931-021-01720-0)
Supplement: Supplementary file 2 — Additional file 2: Predictors of pulmonary function trajectories using alternative linear mixed effects model. [file 12931_2021_1720_MOESM2_ESM.doc]

**TITLE:** Lung Function Trajectories in Children with Post-Prematurity Respiratory Disease: identifying risk factors for abnormal growth

**AUTHORS:**Levin, Jonathan C. MD ^1,2^; Sheils, Catherine A. MD ^2^; Gaffin, Jonathan M. MD MMSc^2^; Hersh, Craig P. MD MPH^3^; Rhein, Lawrence M. MD MPH^4^; Hayden, Lystra P. MD MMSc^2,3^

*Additional File 2. Predictors of pulmonary function trajectories using alternative linear mixed effects model*

|  | **FEV1 % predicted Δ / year** | **FVC % predicted Δ / year** | **FEV1/FVC Δ / year** |
| --- | --- | --- | --- |
| **Neonatal** | | | |
| GA (1 week) | -0.16 (-0.59, -0.16) | 0.42 (-0.29, 0.42) | 0.48 (-0.0053, 0.48) |
| BW (100g) | 0.0021 (-0.29, 0.0021) | 0.13 (-0.3, 0.13) | 0.26 (-0.046, 0.26) |
| SGA | -0.31 (-2.7, -0.31) | 0.67 (-2.4, 0.67) | -1 (-3.4, -1) |
| Multiple gestation | -0.25 (-2, -0.25) | -1.5 (-3.9, -1.5) | 0.25 (-1.6, 0.25) |
| Antenatal steroids (any) | **-3.4 (-6.4, -3.4)*** | 0.93 (-5, 0.93) | -2.1 (-5.6, -2.1) |
| Antenatal steroids (complete) | 0.47 (-1.6, 0.47) | 0.7 (-3.4, 0.7) | 1.6 (-0.65, 1.6) |
| Surfactant | 0.01 (-9.8, 0.01) | 5.1 (-15, 5.1) | -7.6 (-21, -7.6) |
| Ventilated Days (1 day) | **-0.012 (-0.023, -0.012)*** | -0.00035 (-0.014, -0.00035) | **-0.019 (-0.027, -0.019)*** |
| CPAP Days (1 day) | -0.0096 (-0.06, -0.0096) | 0.019 (-0.047, 0.019) | -0.044 (-0.097, -0.044) |
| PDA | 0.48 (-1.7, 0.48) | -1 (-4.1, -1) | -0.41 (-2.5, -0.41) |
| PDA Ligation | -0.1 (-2.5, -0.1) | -0.4 (-3.5, -0.4) | **2.3 (0.071, 2.3)*** |
| NEC | 1.4 (-0.85, 1.4) | 1.2 (-2, 1.2) | 1.2 (-1, 1.2) |
| Severe IVH | -0.17 (-4.7, -0.17) | -1.3 (-6.8, -1.3) | -1.4 (-5.7, -1.4) |
| Postnatal Steroids (in NICU) | -0.64 (-2.2, -0.64) | -0.93 (-4, -0.93) | -0.46 (-2.7, -0.46) |
| Gastrostomy Tube | -0.53 (-2.5, -0.53) | 2.4 (-0.11, 2.4) | -1.6 (-3.5, -1.6) |
| Tracheostomy | -2.5 (-5.1, -2.5) | -0.74 (-4.7, -0.74) | -2.7 (-5.5, -2.7) |
| Discharge Nutrition | -0.64 (-1.8, -0.64) | 0.44 (-1.3, 0.44) | -0.075 (-1.4, -0.075) |
| Any resp support at 36 weeks | -0.17 (-2.4, -0.17) | -0.38 (-3.2, -0.38) | -1.7 (-3.8, -1.7) |
| Any resp support at discharge | -0.1 (-1.8, -0.1) | -0.17 (-2.6, -0.17) | -1.5 (-3.2, -1.5) |
| **Maternal History** | | | |
| Age | -0.0089 (-0.18, -0.0089) | -0.088 (-0.33, -0.088) | -0.075 (-0.22, -0.075) |
| Asthma | -0.84 (-2.5, -0.84) | 0.59 (-2.2, 0.59) | **-1.9 (-3.6, -1.9)*** |
| Eczema | 0.78 (-1.3, 0.78) | 3.4 (-0.089, 3.4) | 1.6 (-0.61, 1.6) |
| Hay Fever | 0.29 (-1.4, 0.29) | 1.7 (-1.4, 1.7) | -1 (-2.9, -1) |
| Atopy | -0.25 (-1.9, -0.25) | 1 (-2, 1) | -0.63 (-2.6, -0.63) |
| Secondhand smoke exposure at home | 0.87 (-1.8, 0.87) | 0.39 (-3.6, 0.39) | 0.6 (-2.3, 0.6) |
| **Follow-up (0-3 year) history (n=42)** | | | |
| Received palivizumab | -3.7 (-10, -3.7) | 2.2 (-10, 2.2) | **-8 (-15, -8)*** |
| Received flu vaccination | -0.5 (-9.1, -0.5) | -1.6 (-14, -1.6) | -0.19 (-11, -0.19) |
| Any ED Visit/Hospitalization | 0.47 (-8.1, 0.47) | **-8.9 (-17, -8.9)*** | 4.6 (-4.2, 4.6) |
| Any hospitalization | **-11 (-22, -11)*** | NA** | -2.8 (-16, -2.8) |
| Any lower respiratory tract infection | 2.5 (-5.7, 2.5) | **-7.9 (-16, -7.9)*** | 3.9 (-4.9, 3.9) |

Multivariable analysis using linear mixed effects model, fixed effects including neonatal exposure, birthweight (BW), gestational age (GA), age, and interaction term of (neonatal exposure * age); random effects including subject and age. Effect β reported is the interaction term of (neonatal effect * age) which represents, controlling for fixed effects above, change in lung function outcome per unit change in risk factor. ***p < 0.05**. ** Not enough degrees of freedom in analysis. PDA = patent ductus arteriosus; NEC = necrotizing enterocolitis. When unit is listed next to risk factor, it indicates unit change for corresponding effect size on spirometry result.
