# Supplementary material for: Lung function trajectories in children with post-prematurity respiratory disease: identifying risk factors for abnormal growth
Source: Respir Res. 2021 May 10;22:143. doi: 10.1186/s12931-021-01720-0 (PMC8112031; doi:10.1186/s12931-021-01720-0)
Supplement: Supplementary file 3 — Additional file 3: Pulmonary function trajectories among studies ≤ 12 years age. [file 12931_2021_1720_MOESM3_ESM.doc]

**TITLE:** Lung Function Trajectories in Children with Post-Prematurity Respiratory Disease: identifying risk factors for abnormal growth

**AUTHORS:**Levin, Jonathan C. MD ^1,2^; Sheils, Catherine A. MD ^2^; Gaffin, Jonathan M. MD MMSc^2^; Hersh, Craig P. MD MPH^3^; Rhein, Lawrence M. MD MPH^4^; Hayden, Lystra P. MD MMSc^2,3^

*Additional File 3. Pulmonary function trajectories among studies ≤ 12 years age*

|  | **FEV1 % predicted (%/yr)** | **FVC % predicted (%/yr)** | **FEV1/FVC (/yr)** |
| --- | --- | --- | --- |
| **Overall** | 2 ± 5.8 | 2.9 ± 6.2 | -1 ± 3.8 |
| **Gender** | | | |
| Male (ref) | 1.8 ± 6 | 3.3 ± 6.1 | -0.37 ± 4.4 |
| Female | 2.2 ± 5.8 | 2.6 ± 6.4 | -1.5 ± 3.3 |
| **Race** | | | |
| Caucasian (ref) | -1.2 ± 6 | 5 ± 6.4 | -2.9 ± 5.2 |
| Black | **2.8 ± 5.7 *** | 2.8 ± 6.6 | -0.34 ± 3.3 |
| Northeast Asian | **-6.9 ± 5.3 *** | 3.3 ± NA | **-12 ± NA *** |
| Other | 1.3 ± 3.6 | 1.8 ± 4.4 | -1.2 ± 1.4 |
| **BPD severity** | | | |
| None/Mild (ref) | 1.5 ± 6.1 | 2.6 ± 6.1 | -1.7 ± 3.8 |
| Moderate/Severe | 3 ± 4.9 | 4.1 ± 6.8 | -0.062 ± 3.8 |

Values represent mean increase ± s.d. *** = p < 0.05.** Significance determined by univariate linear regression. *BPD* Bronchopulmonary Dysplasia, severity defined by modified 2001 NHLBI consensus definition. NA listed for s.d. when only single result available.

*Predictors of pulmonary function trajectories among studies ≤ 12 years age*

|  | **FEV1 % predicted Δ / year** | **FVC % predicted Δ / year** | **FEV1/FVC Δ / year** |
| --- | --- | --- | --- |
| **Neonatal** | | | |
| GA (1 week) | 0.077 (-0.86, 1) | 1 (-0.36, 2.3) | -0.14 (-0.95, 0.67) |
| BW (100g) | 0.26 (-0.39, 0.91) | 0.037 (-0.88, 0.96) | 0.33 (-0.18, 0.85) |
| SGA | -3.1 (-8.3, 2) | -1.3 (-7.7, 5.1) | -2.2 (-6, 1.6) |
| Multiple gestation | -0.58 (-3.3, 2.1) | -3.5 (-7.3, 0.26) | 0.79 (-1.7, 3.2) |
| Antenatal steroids (any) | -1.2 (-5.5, 3.2) | 2 (-7.5, 11) | -2.9 (-7.9, 2.1) |
| Antenatal steroids (complete) | 2.1 (-2.1, 6.3) | 1.7 (-4.6, 8) | 0.92 (-2.3, 4.2) |
| Surfactant | -0.52 (-9.1, 8) | 5.2 (-7.7, 18) | -4 (-11, 3.3) |
| Ventilated Days (1 day) | **-0.027 (-0.046, -0.0091)*** | -0.015 (-0.04, 0.011) | **-0.016 (-0.029, -0.0041)*** |
| CPAP Days (1 day) | -0.0012 (-0.08, 0.078) | 0.05 (-0.053, 0.15) | -0.038 (-0.099, 0.023) |
| PDA | -0.054 (-3.7, 3.6) | **-5.3 (-10, -0.11)*** | 1.7 (-1.3, 4.8) |
| PDA Ligation | -0.93 (-4.1, 2.3) | -2.5 (-6.2, 1.1) | **2.8 (0.61, 5.1)*** |
| NEC | 2.8 (-0.92, 6.5) | 2.5 (-2.2, 7.2) | 1.3 (-1.4, 3.9) |
| Severe IVH | -0.0056 (-7.7, 7.7) | 0.13 (-7.3, 7.6) | 5.8 (-0.61, 12) |
| Postnatal Steroids (in NICU) | -2.6 (-5.9, 0.77) | **-3.7 (-7, -0.37)*** | -1.9 (-5, 1.1) |
| Gastrostomy Tube | **-4.2 (-7.2, -1.1)*** | -2.2 (-5.9, 1.6) | 0.14 (-2.3, 2.6) |
| Tracheostomy | -3.8 (-8.8, 1.3) | -5.3 (-12, 1.6) | -2.9 (-6.8, 0.92) |
| Discharge Nutrition | -0.79 (-2.6, 0.98) | 1 (-1.5, 3.6) | -0.47 (-2, 1.1) |
| Any resp support at 36 weeks | -1.9 (-5.5, 1.6) | 0.14 (-4.5, 4.8) | -1 (-3.9, 1.8) |
| Any resp support at discharge | 0.31 (-2.6, 3.2) | 1.7 (-2.5, 5.9) | -1.9 (-4.5, 0.65) |
| **Maternal History** | | | |
| Age | 0.24 (-0.038, 0.53) | 0.057 (-0.34, 0.46) | 0.061 (-0.16, 0.28) |
| Asthma | **-3.6 (-6.3, -0.94)*** | -1 (-5.2, 3.2) | -2.2 (-4.4, 0.078) |
| Eczema | -0.52 (-4.4, 3.4) | -0.15 (-5, 4.7) | 1.3 (-1.6, 4.3) |
| Hay Fever | -1 (-4.4, 2.4) | -2.6 (-7.1, 1.8) | -1.8 (-4.3, 0.73) |
| Atopy | **-3.5 (-6.2, -0.77)*** | -2.8 (-6.8, 1.2) | -0.81 (-3.2, 1.5) |
| Secondhand smoke exposure at home | 1.9 (-2.9, 6.8) | 1 (-4.9, 6.9) | 0.63 (-2.9, 4.1) |
| **Follow-up (0-3 year) history (n=42)** | | | |
| Received palivizumab | -1.7 (-13, 9.3) | -1.3 (-20, 17) | -4 (-15, 6.5) |
| Received flu vaccination | -2.8 (-12, 6.9) | -0.74 (-15, 13) | -2.8 (-13, 7) |
| Any ED Visit/Hospitalization | 2.1 (-6.4, 11) | -1.5 (-16, 13) | 0.76 (-8.5, 10) |
| Any hospitalization | -11 (-23, 1.7) | -5.3 (-41, 30) | 0.89 (-12, 14) |
| Any lower respiratory tract infection | 5.2 (-3.5, 14) | -10 (-25, 3.8) | 0.74 (-14, 16) |

Multivariable analysis using linear mixed effects model, fixed effects including neonatal exposure, birthweight (BW), gestational age (GA), age, and interaction term of (neonatal exposure * age); random effects including subject and age. Effect β reported is the interaction term of (neonatal effect * age) which represents, controlling for fixed effects above, change in lung function outcome per unit change in risk factor. ***p < 0.05**. ** Not enough degrees of freedom in analysis. PDA = patent ductus arteriosus; NEC = necrotizing enterocolitis. When unit is listed next to risk factor, it indicates unit change for corresponding effect size on spirometry result.
